# The Application of Contrast-Enhanced 3D-STIR-VISTA MR Imaging of the Brachial Plexus

**DOI:** 10.5334/jbsr.2803

**Published:** 2022-09-05

**Authors:** Dingsheng Han, Yanru Zhou, Lan Zhang, Jiajia Zhang

**Affiliations:** 1 Imaging and nuclear medicine Department, Henan University of Chinese Medicine, Zhengzhou, China; 2 MRI Department, the First Affiliated Hospital of Henan University of Chinese Medicine, Zhengzhou, China; 3 Department of Radiology, Gold Coast University Hospital, School of Medicine, Bond University, Australia

**Keywords:** Brachial plexus, Magnetic resonance imaging, 3D-STIR-VISTA, signal to noise ratio, contrast to noise ratio, contrast ratio

## Abstract

**Objective:**

To introduce contrast-enhanced 3D-STIR-VISTA sequence that would improve the image quality for the brachial plexus imaging and enhance the contrast between the brachial plexus and surrounding tissues.

**Methods:**

Thirty subjects (average age, 47.33 ± 15.15 years; 22 males and 8 females) were enrolled, including 7 patients with brachial plexus injuries, 4 patients with schwannomas, 1 patient with neurofibroma, 1 patient with thoracic outlet syndrome, 1 patient with metastasis, 1 patient with brachial plexus neuritis, and 15 patients without abnormal findings. Scores of unenhanced and contrast-enhanced 3D-STIR-VISTA images using a 5-point scale were compared by Wilcoxon’s signed-rank test. The signal intensity (SI), signal to noise ratio (SNR), contrast to noise ratio (CNR) and contrast ratio (CR) between 3D-STIR-VISTA images without and with contrast agent were compared by the paired Student t-test.

**Results:**

The SNRs of the brachial plexus between 3D-STIR-VISTA without and with contrast agent were not significantly different, while SNRs of surrounding tissues were significantly decreased with contrast agent. The CNRs of 3D-STIR-VISTA images with contrast agent were significantly higher than that without contrast agent. The 3D-STIR-VISTA sequence with contrast agent exhibited a statistically higher CR than that without contrast agent. The average score for 3D-STIR-VISTA images with contrast agent was significantly higher than that without contrast agent.

**Conclusion:**

The 3D-STIR-VISTA sequence with contrast agent is qualitatively and quantitatively superior to that without a contrast agent. The contrast-enhanced 3D-STIR-VISTA sequence can provide distinct visualization of the brachial plexus and enhance the contrast between the brachial plexus and surrounding tissues.

## Introduction

The brachial plexus is a complex network of nerves, formed by the anterior rami of the C5~8 cervical nerves and T1 nerve, which can be divided into pre-ganglionic and post-ganglionic parts [1]. Owing to the deep location of the brachial plexus and its complex anatomic architecture, the lesions of the brachial plexus are difficult to diagnose, characterize and treat [2]. So far, the advantage of magnetic resonance imaging (MRI) in the diagnosis of brachial plexus is conspicuous [3]. The MRI can provide detailed anatomic information, known as a non-invasive imaging modality with high soft tissue contrast, allowing multi-plane and multi-angle observation of the brachial plexus [4,5].

On unenhanced T1-weighted imaging (T1WI), the signals of the brachial plexus are similar to those of the surrounding muscles and soft tissues while it has a relatively high signal compared with adjacent muscles and fat on T2-weighted imaging (T2WI). Whether on T1WI or T2WI, the contrast between the brachial plexus and surrounding tissue is suboptimal [6]. Currently, there are several MRI protocols for visualization and evaluation of the brachial plexus. One of the most frequently utilized approaches is diffusion weighted imaging with background suppression (DWIBS) [7]. DWIBS has the advantage of providing adequate contrast between the brachial plexus and surrounding tissues compared with conventional T1WI and T2WI. However, DWIBS fails to accurately reflect architecture of the brachial plexus due to the low spatial resolution.

DWIBS is inferior in depiction of preganglionic brachial plexus and is also poor in the clear visualization of the brachial plexus in patients with regional lymph nodes because lymph nodes typically demonstrate similar high signals with nerves [8,9,10]. Another standard MRI protocol for assessing the brachial plexus is short inversion time inversion recovery T2-weighted imaging (STIR-T2WI). Limitations of STIR-T2WI sequence include increased minimal time of repetition (TR), increased acquisition time and reduced signal to noise ratio (SNR) [11,12,13]. The three-dimensional spin echo–type isotropic imaging is performed using the sampling perfection with application optimized contrasts using varying flip angle evolutions (SPACE, Cube and VISTA) imaging techniques [14]. With a Philips 3.0T MRI scanner, a 3D-STIR-VISTA (three-dimensional short inversion time inversion recovery volumetric isotropic turbo spin echo acquisition) imaging technique can maintain a good SNR and an adequate contrast within a reasonable acquisition time, with a short STIR preparation added to guarantee homogeneous fat saturation [14]. As a consequence, brachial plexus could be better delineated by 3D-STIR-VISTA sequence. However, it still has limited capacity in displaying the brachial plexus due to hyperintense vessels or lymph nodes and insufficient contrast between nerves and surrounding tissues [14,15]. One well-known problem with 3D-STIR-VISTA sequence is overlapping with high signal from veins, which renders the 3D visualization of the brachial plexus somewhat difficult.

The distinct display of the brachial plexus cannot be realized without enhancing the contrast between the nerves and surrounding tissues. The initial motivation underlying this study was to enhance the contrast between nerves and surrounding tissues by combining 3D-STIR-VISTA with a sufficient background signal suppression. The paramagnetic contrast agent-gadolinium chelate is known to have not only a T1- shortening effect, but also T2-shortening effect. Therefore, it can darken the area of contrast distribution depending on its concentration on the T2WI [16]. Due to the T2-shortening effect, 3D-STIR-VISTA sequence with the administration of gadolinium could provide a clear-cut outlines of the brachial plexus with high resolution. We compared 3D-STIR-VISTA images without and with contrast agent to evaluate the improvement of image quality and the contrast between the brachial plexus and surrounding tissues.

## Materials and Methods

### Subjects

From January 2017 to June 2021, a total of 30 subjects with brachial plexus MRI were recruited in this study. There were 22 males and 8 females, and the mean age was 47.33 ± 15.15 years (ranging from 16 to 57 years), including 7 patients with brachial plexus injuries, 4 patients with schwannomas, 1 patient with neurofibroma, 1 patient with thoracic outlet syndrome (TOS), 1 patient with metastasis, 1 patient with brachial plexus neuritis, and 15 patients without abnormal findings. The clinical symptoms of the patients included shoulder and upper limb pain, numbness, dysfunction and amyotrophy.

### MRI protocols

A MRI scan was performed on a 3-Tesla MRI scanner (Ingenia, Philips Healthcare, Best, Netherlands) with a 32-channel body coil. The standard imaging protocols included T1WI and STIR-VISTA in the coronal and transverse planes. The 3D-STIR-VISTA parameters were as follows: TR/TE = 3400/220 ms, TI = 220 ms, slice thickness = 1.5 mm, flip angle = 180°, field of view (FOV) = 400 × 384 mm, matrix = 384 × 384, NSA = 1.8, slice per slab = 60. The acquisition time for every scan was 8mins 30s. After plain scan, the contrast-enhanced scan was performed immediately by intravenous injection of contrast agent (Gd-DOTA, 0.2 ml/kg, Heng Rui Pharmaceutical Co, China) at a flow rate of 2.5 mL/s followed by a 20-mL saline flush at the same rate. The acquisition time for the total imaging was 17mins.

### Image evaluation

Raw images were transferred to the work station and the maximum intensity projection (MIP) was constructed. The regions of interest (ROIs) (about 20 pixels) were marked on the bilateral C5–C7 brachial plexus (two cm away from the thecal sac edge). Regarding the surrounding tissues, the ROIs were drawn close to the brachial plexus. Two senior radiologists measured the average signal intensity (SI) and mean square deviation (SD) of the brachial plexus and surrounding tissues. The signal to noise ratio (SNR), contrast to noise ratio (CNR) and contrast ratio (CR) between the brachial plexus and surrounding tissues were calculated according to the equations:

1. SNR = SI nerve or SI tissue/SD tissue
2. CNR = (SI nerve-SI tissue)/SD tissue
3. CR = (SI nerve-SI tissue)/(SI nerve + SI tissue)

In order to evaluate the image quality of 3D-STIR-VISTA images with and without contrast agent, a 5point scale was obtained by two experienced radiologists based on nerve visibility, tissue contrast and edge sharpness as follows: 5 = excellent, 4 = good, 3 = fair, 2 = poor, and 1 = uninterpretable. A score of five points was considered excellent for tissue contrast and edge sharpness with continuous and clear visualization of the brachial plexus whereas one point was considered very poor for visualization of brachial plexus with serious image blurring.

### Statistical analysis

Statistical analyses were performed with SPSS Statistical Solutions (version 22.0, IBM Corporation, New York, USA). The paired Student t-test was used to assess the differences in SI, SNR, CNR and CR between 3D-STIR-VISTA images without and with contrast agent. Wilcoxon’s signed-rank test was used to compare the scores between 3D-STIR-VISTA images and contrast-enhanced images. P-value < 0.05 was considered statistically significant.

## Results

The 3D-STIR-VISTA images and contrast-enhanced images were successfully acquired in all patients. The surrounding tissues demonstrated low signals with the contrast of hyperintense brachial plexus, while the trunks, divisions and cords of the brachial plexus were indistinct because the small veins, lymph nodes and other surrounding tissues overlapped in the 3D-STIR-VISTA images. After the administration of contrast agent, the contrast-enhanced 3D-STIR-VISTA images showed the signals of vessels, lymph nodes and other surrounding tissues were suppressed and signal of the brachial plexus was relatively increased. The roots, trunks, divisions and cords of brachial plexus could be continuously displayed.

**Figure 1:**
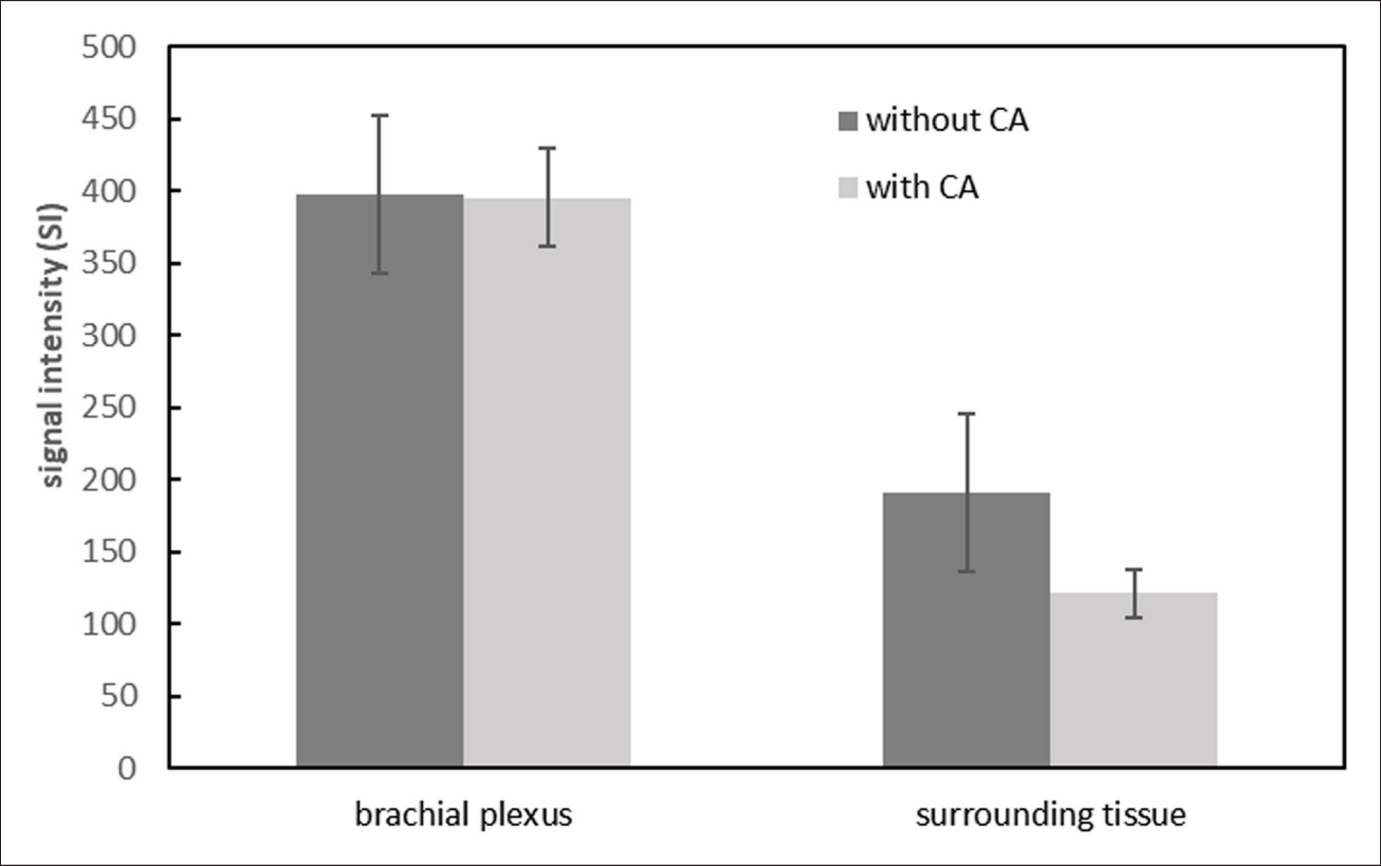
SIs of surrounding tissues were significantly decreased after contrast agent, whereas that in the brachial plexus was not.

**Figure 2:**
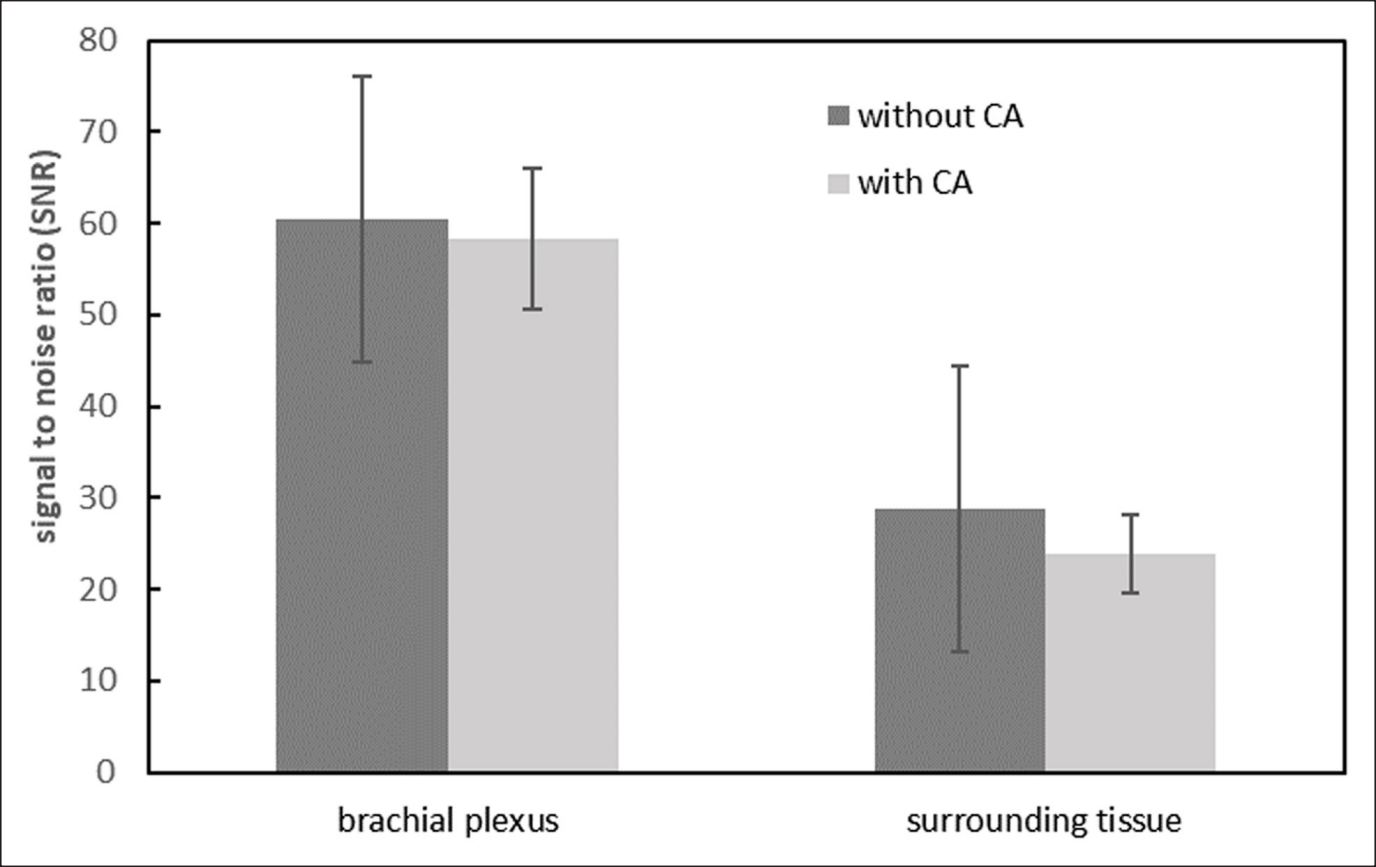
SNRs of surrounding tissues were significantly decreased after contrast agent, whereas that in the brachial plexus was not.

**Figure 3:**
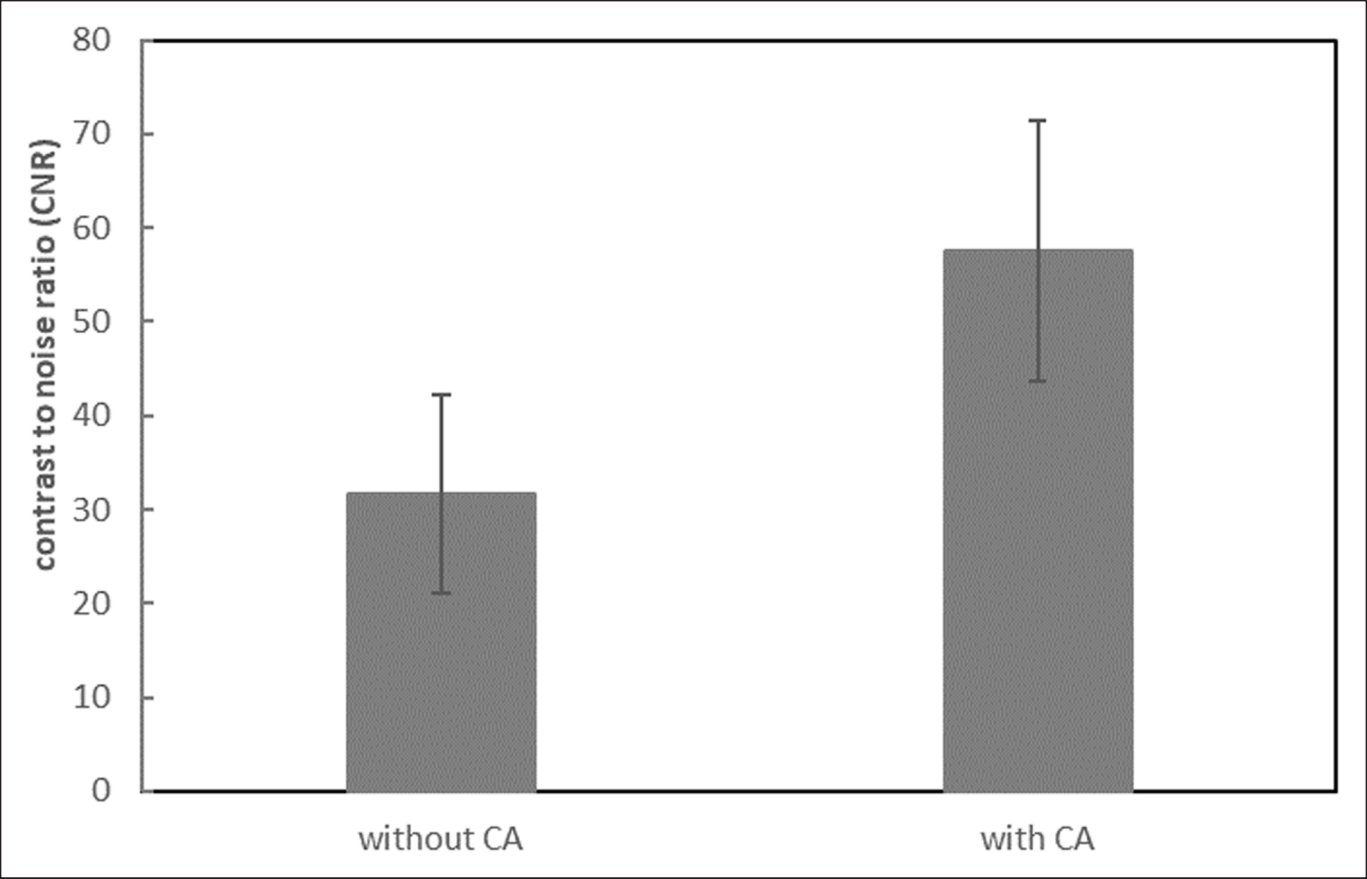
CNR was significantly increased after contrast agent in the 3D-STIR-VISTA sequence.

**Figure 4:**
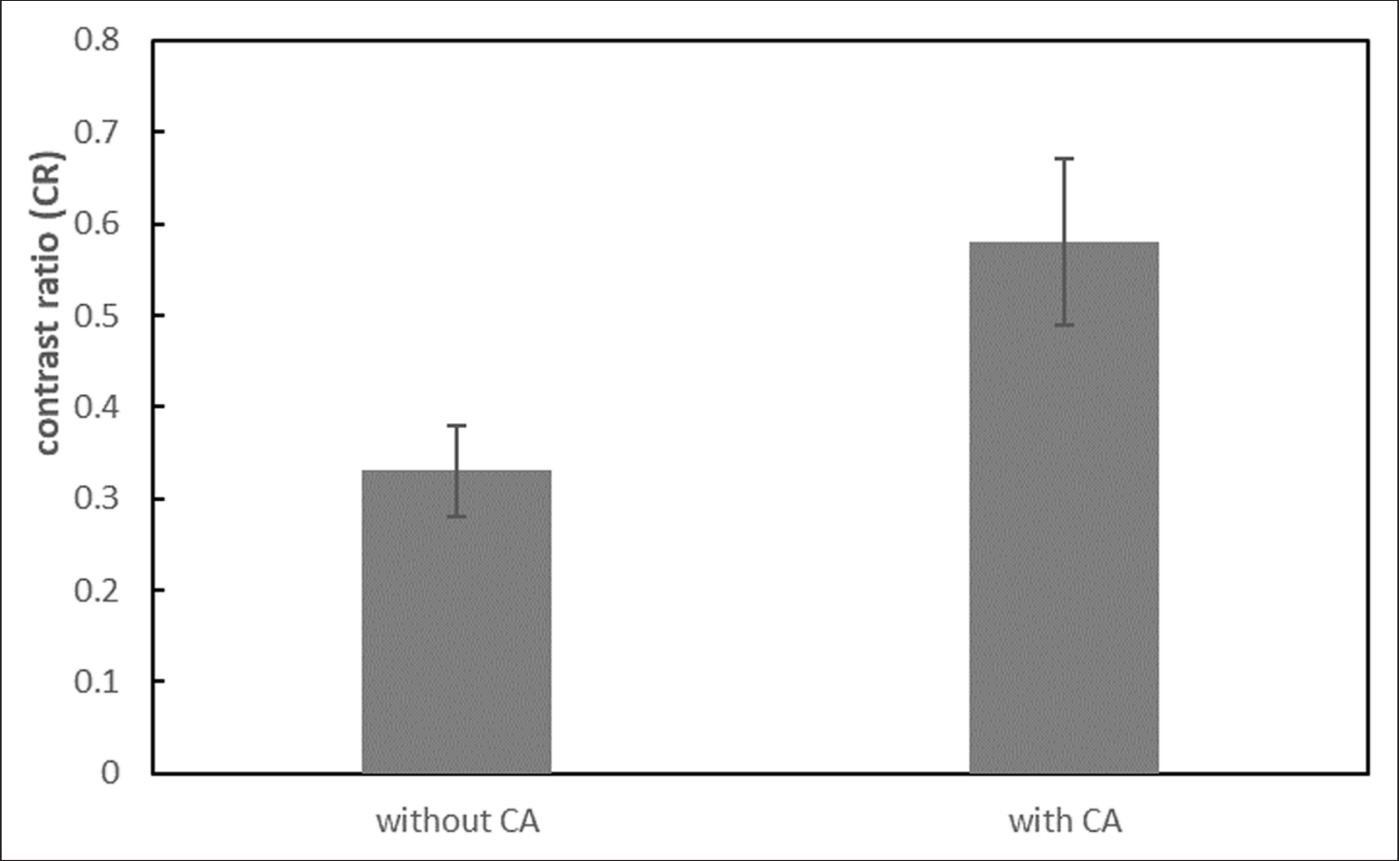
CR was significantly increased after contrast agent in the 3D-STIR-VISTA sequence.

The SIs of the brachial plexus between 3D-STIR-VISTA without and with contrast agent were not significantly different, however, the SIs of surrounding tissues were significantly suppressed with contrast agent (Table 1, Figure 1). The SNRs of the brachial plexus between 3D-STIR-VISTA without and with contrast agent were not significantly different, while SNRs of surrounding tissues were significantly decreased with contrast agent (Table 1, Figure 2). Comparison of CNRs between unenhanced and contrast-enhanced 3D-STIR-VISTA indicated that contrast-enhanced 3D-STIR-VISTA sequences showed a statistically higher CNR (Table 1, Figure 3). The 3D-STIR-VISTA sequence with contrast agent exhibited a statistically higher CR than that without contrast agent (Table 1, Figure 4).

**Table 1:** Comparisons of SI, SNR, CNR and CR between 3D-STIR-VISTA without and with contrast agent (CA).


	IMAGES WITHOUT CA	IMAGES WITH CA	P VALUE

SI of brachial plexus	397.51 ± 58.76	395.16 ± 54.89	0.873

SI of surrounding tissue	190.69 ± 33.94	120.94 ± 16.54	<0.001

SNR of brachial plexus	60.51 ± 16.59	58.27 ± 15.65	0.593

SNR of surrounding tissue	28.86 ± 7.66	23.91 ± 4.34	<0.001

CNR	31.69 ± 10.6	57.54 ± 13.87	<0.001

CR	0.33 ± 0.05	0.58 ± 0.09	<0.001


**Figure 5:**
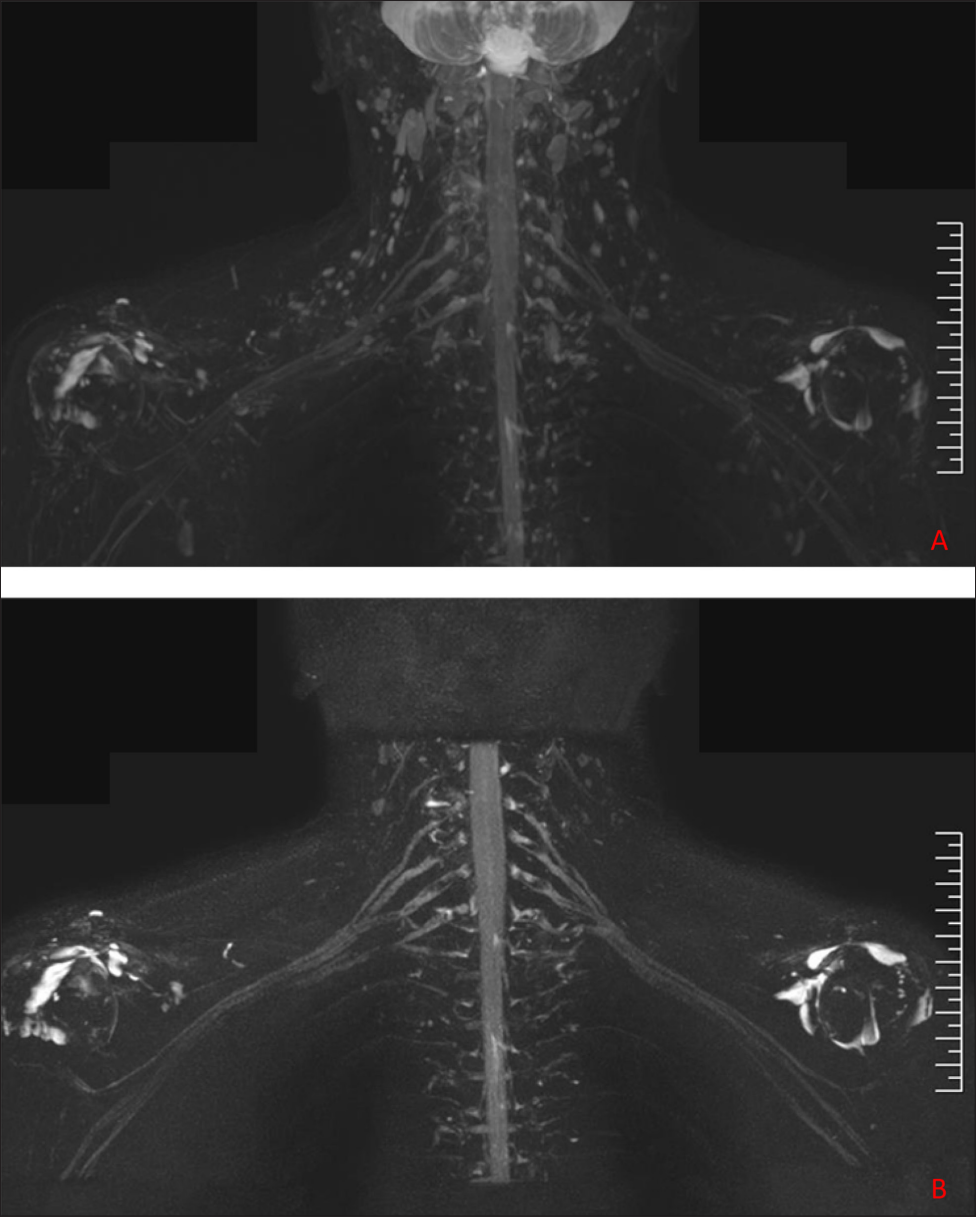
3D-STIR-VISTA **(A)** and contrast-enhanced image **(B)** of normal brachial plexus. A.) The boundary of the brachial plexus was not very clearly visualized due to interference by the veins, lymph nodes and other surrounding tissues. B.) The signals of adjacent muscles, veins, lymph nodes were suppressed on the contrast-enhanced images. Outlines of the brachial plexus became sharp, and the roots, trunks, divisions and cords could be completely and continuously displayed.

**Figure 6:**
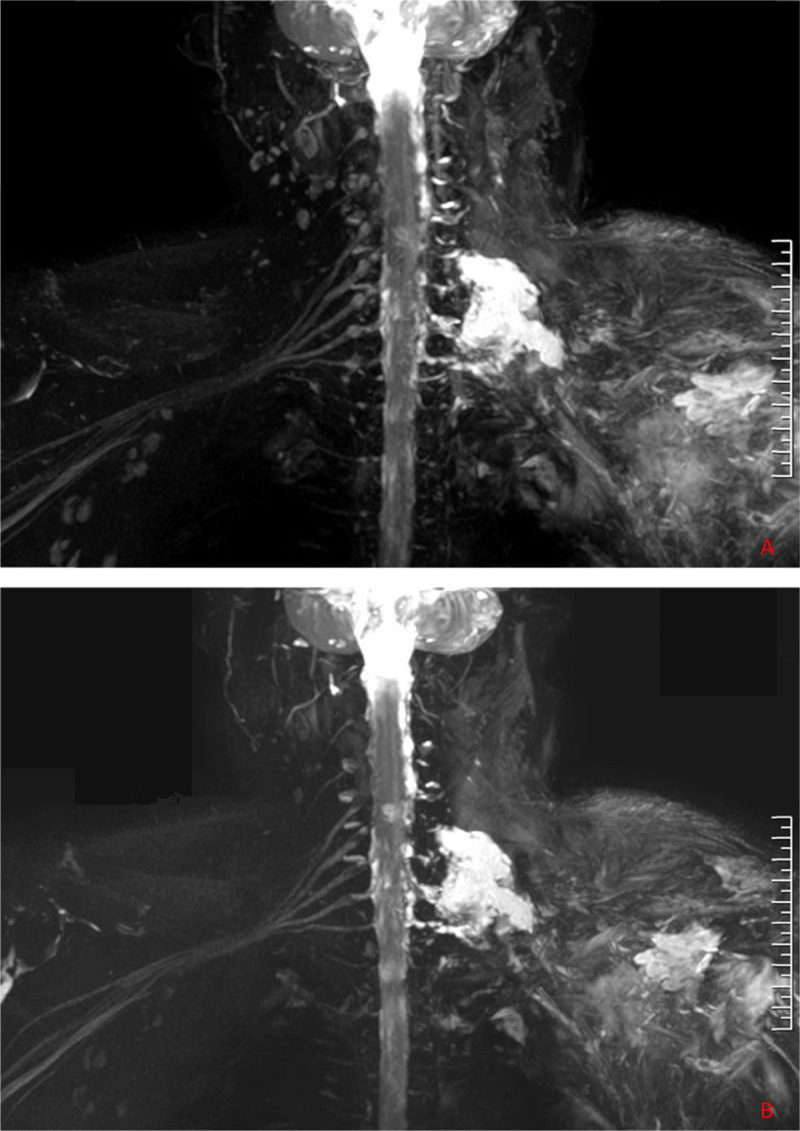
3D-STIR-VISTA image **(A)** and contrast-enhanced image **(B)** of brachial plexus injury. A.) 3D-STIR-VISTA showed the soft tissue of left shoulder got serious contusion and swollen, and the continuity of C5~8 brachial plexus was lost. B.) The contrast-enhanced 3D-STIR-VISTA clearly showed the damage of the brachial plexus with increased and discontinuous signals.

The mean scores were 2.5 ± 0.57 and 4.8 ± 0.41 for 3D-STIR-VISTA images without and with contrast agent, respectively (Table 2). The higher score of contrast-enhanced 3D-STIR-VISTA images indicated the image quality would be improved greatly compared with unenhanced 3D-STIR-VISTA images (P < 0.001), as shown in Figure 5 for the normal brachial plexus, and Figure 6 for brachial plexus injury.

**Table 2:** Scores for 3D-STIR-VISTA images without and with contrast agent (CA).


SCORE	IMAGES WITHOUT CA	IMAGES WITH CA

5	0	24

4	0	6

3	15	0

2	14	0

1	1	0

Total	30	30

Mean	2.5 ± 0.57	4.8 ± 0.41


## Discussion

The aim of our study is to improve image quality and CR of the brachial plexus using contrast-enhanced 3D-STIR-VISTA sequence, in which an advanced fat-suppressed T2WI with high resolution and a strong background signal suppression are used to integrally delineate the architecture of the brachial plexus and increase the contrast between the brachial plexus and surrounding tissues. With enhanced 3D-STIR-VISTA sequence, the roots, trunks, divisions, cords and branches of the brachial plexus are clearly visualized at different levels. It is much easier to analyze the spatial localization of the brachial plexus within and along nerve segments. Our results show that contrast-enhanced 3D-STIR-VISTA can provide high spatial resolution, large scale of FOV, excellent contrast and continuous contours of the brachial plexus.

Since the brachial plexus is located between fat and muscles, suppression of signals from fat, muscles, veins and other tissues in the background is indispensable for an ideal display of the brachial plexus. A STIR TSE sequence utilizing frequency selective fat saturation is more satisfactory to visualize the contours, architecture and continuity of the brachial plexus, as well as its relationship to the surrounding lesions and musculoskeletal structures [17,18]. With current Philips 3.0T MRI scanner, 3D-STIR-VISTA sequence is a STIR TSE sequence in conjunction with an adiabatic T2 and motion sensitized preparation based on improved motion-sensitized driven equilibrium (iMSDE) for brachial plexus imaging [19]. The iMSDE prepulse results in uniform arterial and venous signal suppressions, mainly signals from the subclavian arteries and veins, enhancing the visualization of the nerve structures. However, it is very weak in the suppression of small veins and lymph nodes in the background. In order to further improve the brachial plexus imaging, 3D-STIR-VISTA coupled with contrast agent is adopted in a clinical routine setting, thereby enhancing the contrast between the nerves and surrounding tissues by completely inhibiting the high signals from vessels and lymph nodes in the background. Gadolinium is a kind of paramagnetic contrast agent with function in shortening both T1 and T2 relaxation time [20]. We take advantage of T2 shortening effect of gadolinium on 3D-STIR-VISTA sequence combined with suppressing the signals from contrast-containing fat, muscles, bones, and veins in the background. Gadolinium cannot enter the normal nervous tissues due to the blood-nerve barrier [21]. On the contrast-enhanced 3D-STIR-VISTA images, the signals from the normal brachial plexus are not affected while high signals from vessels are completely suppressed; thus, the contrast between the brachial plexus and surrounding tissues is strengthened. Moreover, the contrast-enhanced 3D-STIR-VISTA will provide comprehensive brachial plexus assessment especially in patients with severe pain or claustrophobia who cannot tolerate an over 20-minute MRI examination. This is particularly important in patients with neurological disturbances due to a brachial plexus injury.

CNR reflects the difference in the SNR between two tissues, while CR represents the relative difference in signals from different tissues [22]. Also CR of contrast-enhanced 3D-STIR-VISTA images greatly improves, owing to the further suppression of vessels, lymph nodes and other surrounding tissues. The average score for image quality of 3D-STIR-VISTA images with contrast agent is much higher than that without contrast agent. Furthermore, the grading for contrast-enhanced 3D-STIR-VISTA images is mostly five or four points due to the prominent visualization of the brachial plexus and remarkable contrast between the brachial plexus and surrounding tissues.

In the previous literatures, 3D-STIR SPACE sequence (Sampling Perfection with Application optimized Contrasts using different flip angle Evolution) with the administration of gadolinium has been frequently used for the brachial plexus imaging [7,23]. However, 3D-STIR-VISTA sequence has not been fully discussed before. Our study had several limitations including its small sample size (a total of 30 patients with lesion or normal brachial plexus). And some brachial plexus injuries were managed conservatively and the radiologic findings could not be confirmed by surgery. Moreover, it is difficult to distinguish terminal branches of the brachial plexus (musculocutaneous nerve, axillary nerve, radial nerve, median nerve and ulnar nerve) with the current imaging resolution. In addition, overall evaluation for the brachial plexus could not merely rely on a solo 3D-STIR-VISTA sequence, and other routine sequences still need to be integrated.

## Conclusion

In summary, the contrast-enhanced 3D-STIR-VISTA utilizing T2-shortening effect of gadolinium, which is iMSDE T2WI TSE sequence with a fat saturation, can enhance the contrast between the brachial plexus and surrounding tissues. The 3D-STIR-VISTA sequence with contrast agent is qualitatively and quantitatively superior to that without contrast agent. It can provide distinct visualization and accurate assessment of anatomies and pathologies of the brachial plexus. We believe the contrast-enhanced 3D-STIR-VISTA will play a major role in the work-up of the brachial plexus imaging, substantially improving early diagnosis and management for patients with brachial plexopathy.
